# An Implementation Model for Oncofertility Support in a Regional Cancer Center without an In-House Reproductive Medicine Unit

**DOI:** 10.24546/0100505581

**Published:** 2026-08-31

**Authors:** MAHO AZUMI, ERI OKAMOTO, AKIKO UCHIDA, MASAHIDE SHIOTANI, YOSHITO TERAI

**Affiliations:** 1 Department of Obstetrics and Gynecology, Kobe University Hospital, Kobe, Japan; 2 Hanabusa Women’s Clinic, Kobe, Japan

**Keywords:** Oncofertility, Fertility preservation, Implementation model, Multidisciplinary care, External collaboration, Supportive cancer care

## Abstract

**PURPOSE:**

Fertility loss related to cancer treatment can significantly affect quality of life among pediatric, adolescent, and young adult patients. Many regional cancer centers lack an in-house reproductive medicine unit, which creates barriers to timely fertility preservation (FP). To address this gap, our hospital established a multidisciplinary Oncofertility Working Group in collaboration with nearby reproductive centers. This study evaluated referral activities and outcomes after working group establishment and aimed to propose a feasible FP support implementation model for cancer centers without reproductive medicine units.

**METHODS:**

We developed a standardized referral pathway and consultation checklist sheet in collaboration with a primary external reproductive center. We retrospectively analyzed consecutive patients referred for FP using consultation checklist sheets and medical records. Questionnaire surveys were conducted at working group launch and 4 years later to assess institutional changes.

**RESULTS:**

Between April 2022 and September 2025, 45 patients (24 female and 21 male) were referred for FP. The median interval from referral to reproductive consultation was 2 days. FP was performed in 70.8% of female patients and 95.2% of male patients. Survey results showed a shift from department-dependent practices to a standardized referral system.

**CONCLUSION:**

Even in a regional cancer treatment cooperation base hospital without an in-house reproductive medicine unit, multidisciplinary coordination and structured external collaboration can deliver timely and practical FP support. This implementation model may help reduce regional disparities in oncofertility care, although further work should strengthen information sharing and long-term follow-up.

## INTRODUCTION

Cancer treatment-related loss of fertility can substantially reduce quality of life among young patients with cancer (1–4). Improvements in cancer survival have increased attention to survivorship and long-term life planning, and fertility preservation (FP) has become an essential component of supportive care for pediatric, adolescent, and young adult populations (3, 5). International guidelines, including those from the American Society of Clinical Oncology and the European Society of Human Reproduction and Embryology, recommend counseling about fertility risks and preservation options before initiation of gonadotoxic therapy, along with timely referral to reproductive specialists as part of standard care (6–9). However, routine clinical implementation remains inconsistent. Previous studies report wide variability in awareness, referral patterns, and institutional readiness, which creates a persistent gap between guideline recommendations and actual care delivery (10, 11).

In Japan, collaboration between oncology and reproductive medicine has developed gradually through national and regional initiatives (12, 13). However, implementation varies widely across institutions. Facilities without in-house reproductive medicine services often rely on individual departments or physicians to provide FP support, which can limit access to appropriate counseling and timely referral. Covelli et al. reported that limited access to FP options, poorly established referral pathways, and weak connections between clinicians and fertility clinics reduce the frequency of fertility discussions and referrals (14). To address these barriers, multidisciplinary teams, working groups (WGs), and network-based models have been proposed to support systematic implementation of oncofertility services through structured external collaboration rather than individual effort alone (11, 15–18).

Our hospital is a designated regional cancer treatment cooperation base hospital, but it does not have an in-house reproductive medicine unit and cannot perform sperm, oocyte, or embryo cryopreservation for patients seeking FP. Clarifying the current status of oncofertility care at our institution, establishing a referral pathway suited to local resources, strengthening collaboration with fertility clinics, and making referral practices transparent therefore represent key institutional priorities.

## MATERIALS AND METHODS

In 2021, we established a multidisciplinary Oncofertility Working Group composed of healthcare professionals from multiple clinical departments and initiated development of an FP counseling and referral framework in collaboration with a nearby reproductive center. At the time of WG establishment, we conducted a self-administered questionnaire survey among physicians involved in cancer care at our hospital to assess current practices and needs related to FP and oncofertility care. Patients were referred, in principle, to a partner reproductive center with which our hospital had an established collaboration. During the first six months, we developed standardized referral tools, including referral flowcharts (Figure 1) and a Consultation Checklist Sheet (CCS; Figure 2).

We retrospectively collected referral and cryopreservation outcomes from CCS records and electronic medical records. Collected variables included CCS use, interval from referral to initial consultation in days, age, sex, marital status, pregnancy and childbirth history, number of children for male patients, live birth after treatment, survival status, recurrence, referring department, primary diagnosis, initial versus recurrent disease status, gonadotoxicity risk, cryopreservation type and implementation status, ovulation induction method, and cryopreservation outcomes. We report continuous variables as means or medians and categorical variables as counts and percentages. Statistical analyses were performed using EZR version 1.66 and Microsoft Excel.

Four years after WG establishment, in 2025, we conducted an anonymous questionnaire survey among WG members using Google Forms to obtain qualitative feedback on WG activities and remaining challenges.

## RESULTS

### Initial questionnaire surveys

At the launch of the WG in July 2021, we conducted a questionnaire survey among six departments frequently involved in oncofertility care at our hospital: hematology/oncology, urology, pediatrics, breast surgery, obstetrics and gynecology, and orthopedic surgery. All departments responded, yielding a 100% response rate (Figure 3). Responses showed substantial interdepartmental differences regarding whether physicians had been consulted by patients about sperm, oocyte, or embryo cryopreservation before cancer treatment. Urology reported frequent patient-initiated consultations, whereas hematology/oncology and pediatrics more often indicated that clinicians provided fertility information proactively rather than in response to patient inquiries.

Regarding whether physicians explained FP options to young patients with cancer, urology reported a high explanation rate. Pediatric respondents stated that fertility-related counseling was routinely provided to all eligible patients, reflecting a department-specific counseling policy. With respect to collaboration with reproductive medicine institutions, urology and hematology/oncology reported established partnerships with specific reproductive centers. In contrast, breast surgery, obstetrics and gynecology, pediatrics, and orthopedic surgery reported no fixed affiliations and relied mainly on case-by-case referrals.

Free-text responses identified several key issues. First, sperm cryopreservation is relatively straightforward when ejaculation is possible, which highlights the value of rapid referral to established partner centers. Second, continued fertility counseling within pediatric clinics is considered important for patients younger than 18 years with osteosarcoma or Ewing sarcoma. Third, in acute myeloid leukemia, limited time before treatment initiation often prevents ovarian cryopreservation, although counseling about ovarian preservation before hematopoietic stem cell transplantation remains important. These findings informed subsequent WG discussions and guided development of standardized referral pathways.

### Patient characteristics and referral outcomes

Between April 2022 and September 2025, 45 patients (24 females and 21 males) were referred for FP. Median age measured 29 years for females with a range of 16–42 years and 24 years for males with a range of 9–47 years (Table I). Most referrals occurred at initial diagnosis (42 patients, 93.3%), whereas three patients (6.7%) were referred at recurrence. Nine patients (20.0%) were married, and five patients (11.1%) had one to three children. The CCS was used in 37 cases (82.2%; Table II). In two cases (4.4%), the CCS was completed after the counseling visit. One to two referrals per year were made without CCS completion despite WG.

The median interval from referral to the first reproductive medicine visit was 2 days among patients referred to the primary collaborating reproductive center. Most consultations occurred within 0–3 days after referral with four same-day visits, 10 at 1 day, seven at 2 days, and five at 3 days. A small number required more than 10 days because of clinical or patient-related factors, and referral dates were unavailable in several cases. Ten departments generated referrals, which indicates broad multidisciplinary participation across the hospital.

Hematologic malignancy represented the most common diagnosis with 13 patients (28.9%). Bone and soft tissue cancers followed with six patients (13.3%), breast cancer with five patients (11.1%), and testicular and head and neck cancers with four patients each (8.9%; Table III). Autoimmune disease occurred in three patients (6.7%), and brain tumors in two patients (4.4%). Colorectal, lung, skin, ovarian, prostate, and pancreatic cancers, as well as aplastic anemia, each occurred in one patient (2.2%). Based on planned treatment, gonadotoxicity risk was classified as high in 12 cases (26.7%), intermediate in 17 cases (37.8%), low in nine cases (20.0%), unknown in six cases (13.3%), and not applicable in one case (2.2%; Table IV).

Among female patients, 17 of 24 (70.8%) underwent FP, with seven patients (29.2%) receiving counseling only without cryopreservation. One counseling-only patient later achieved a live birth through assisted reproductive technology at another institution; this patient had a history of acute myeloid leukemia treated more than 10 years earlier. Oocyte cryopreservation accounted for 15 of 17 female FP cases (88.2%), and embryo cryopreservation for two cases (11.8%). Controlled ovarian stimulation used antagonist protocols in 10 cases (58.8%), antagonist plus aromatase inhibitor in one case (5.9%), progestin-primed ovarian stimulation in five cases (29.4%), clomiphene citrate in one case, and in vitro maturation in one case (5.9%); clomiphene citrate and in vitro maturation were used in the same patient. Thirteen female patients (76.5%) completed one stimulation cycle, and four patients (23.5%) completed two cycles. The mean number of cryopreserved oocytes was 8.2 per cycle with a range of 0–28 and 10.8 per patient with a range of 1–28.

Among male patients, 20 of 21 (95.2%) underwent FP, with one patient receiving counseling only. All FP procedures in male patients involved sperm cryopreservation. Seventeen patients (85.0%) completed one collection cycle, and three patients (15.0%) completed two cycles. The mean number of cryopreserved vials was 5.3 per cycle with a range of 0–14 and 6.1 per patient with a range of 0–14. Table V summarizes details of FP performance, cryopreservation type, stimulation method, and outcomes.

Follow-up data were available for all 45 patients. At last follow-up, 37 patients (82.2%) were alive, four patients (8.9%) had experienced recurrence, one patient (2.2%) had died from the primary disease, and three patients (6.7%) had transferred care to other hospitals (Table VI).

### Follow-up questionnaire survey

In August 2025, we conducted an anonymous questionnaire survey among 15 WG members and received responses from 10 members, yielding a response rate of 66.7% (Figure 4). When asked whether WG participation helped establish oncofertility systems within their departments, 30% of respondents selected “extremely helpful” and 50% selected “moderately helpful,” for a combined positive rating of 80%. No respondent selected “not helpful at all,” and 20% selected “neither helpful nor unhelpful.” Regarding whether the WG made interdepartmental collaboration on FP easier, 20% answered “very much” and 30% answered “a little.” In contrast, 40% selected “cannot say either way,” and 10% selected “not much,” indicating variability in perceived impact. Most respondents (70%) reported active use of WG tools such as consultation checklists and referral flowcharts in daily practice. The remaining 30% reported no use, mainly because they were non-physician staff or had not encountered eligible patients. All respondents rated the meeting frequency of once every six months as appropriate. No respondent indicated that meetings were too frequent or too infrequent. For meaningful WG activities with multiple answers allowed, “case-based feedback” ranked highest at 33%, followed by “case sharing across departments” at 25%, “sharing recent FP knowledge” at 21%, and “discussion of practical improvements” at 21%.

Free-text suggestions for future WG activities included organizing seminars and lectures, developing and sharing patient education materials and pamphlets, increasing oncofertility awareness inside and outside the hospital, presenting WG activities at academic meetings such as the Japan Society of Cancer and Reproductive Medicine, evaluating pregnancy and childbirth outcomes, and obtaining expert input on advanced topics such as preimplantation genetic testing for monogenic disorders. Regarding transferability of the WG model to other institutions, 50% answered “yes” and 50% answered “unsure,” whereas no respondent selected “difficult.” Supportive comments highlighted feasibility, value of structured collaboration, and benefits of case and knowledge sharing. More cautious comments noted that feasibility may depend on institutional size, number of participating departments, and available staffing, particularly in community hospitals.

## DISCUSSION

The initial survey conducted in 2021 showed substantial interdepartmental variability in oncofertility counseling and referral practices, with most approaches driven by individual physician experience. The survey clarified the baseline status of oncofertility care at our institution and identified key operational gaps. These findings supported development of a structured and feasible support framework tailored to local needs. Because oncofertility practice varies widely across institutions, local assessment of workflows and barriers remains essential for building an effective and practical support system. To encourage participation from multiple professions and clinical departments, the director of the department personally approached the leaders of each relevant division and requested their involvement. Securing commitment at the leadership level facilitated the recruitment of participants from a wide range of professions and specialties. However, the degree of engagement during meetings varied among participants and appeared to depend, at least in part, on the individual representative’s level of interest in oncofertility care. The 2025 follow-up survey showed broader dissemination and practical use of standardized referral flowcharts and consultation checklists. Eighty percent of WG members rated the WG as helpful for establishing oncofertility systems, and 70% reported routine use of standardized tools in daily practice. These results suggest that the WG promoted a shared institutional framework and common operational approach to oncofertility support across departments, in line with prior reports that multidisciplinary models strengthen oncofertility implementation (19, 20).

This study shows that institutions without an in-house reproductive medicine unit can still establish a rapid and reliable FP support system through structured collaboration with nearby reproductive centers and multidisciplinary coordination. The median referral-to-visit interval of 2 days holds clear clinical relevance because it allows FP options to proceed without delaying cancer treatment initiation (21). Weekend service availability and geographic proximity of the collaborating reproductive center likely supported timely referral and counseling. Several operational challenges nevertheless emerged. Feedback reports were occasionally unavailable when the designated reproductive specialist at the partner center was absent. In addition, physicians with limited oncofertility experience sometimes selected suboptimal ovarian stimulation protocols, which resulted in lower-than-expected oocyte yield.

Standardized referral pathways and consultation checklist use reduced dependence on individual physician practices. Checklist-based standardization has improved quality and reproducibility across many patient safety and quality improvement settings (22). Checklists completed by the referring physician allowed reproductive specialists to plan stimulation protocols, evaluate feasibility, and confirm infection status in advance. Inclusion of subsidy-related items also reduced administrative workload by limiting repeated inter-institutional communication. The Japan Society for Fertility Preservation also provides a standardized CCS to facilitate communication between oncology and reproductive medicine providers (23). Although the content of our institutional CCS is broadly similar to this standardized form, several elements are not currently included, such as eligibility for public financial support programs, the treating physician’s recommendation regarding fertility preservation, the patient’s expectations and preferences, and considerations related to psychosocial support. If necessary, the content of our CCS may be revised and refined in the future to better meet local needs and further facilitate efficient interdisciplinary communication and referral. Nevertheless, referrals without checklist completion continued even 4 years after implementation, highlighting the need for ongoing education and reinforcement. Frequent staff turnover at university hospitals requires continuous information sharing within each department. Our WG therefore asks each department to maintain at least one designated representative and to appoint a successor when personnel changes occur.

National and prefectural oncofertility frameworks now exist, but awareness among hospitals, departments, and individual healthcare providers remains uneven (24, 25). Institutional hard infrastructure alone does not guarantee timely cryopreservation and treatment initiation; effective local operational processes and coordination are also required. In Japan, Gifu Prefecture pioneered a prefecture-wide oncofertility network (“the Gifu Model”), which established close collaboration among oncologists, reproductive specialists, healthcare professionals, and local government agencies to facilitate timely fertility counseling and fertility preservation for adolescent and young adult cancer patients. Furui et al. reported that this regional network successfully improved access to oncofertility services and highlighted the importance of structured interinstitutional collaboration in supporting fertility preservation decision-making (26). The Gifu Model subsequently became one of the representative frameworks for regional oncofertility care in Japan. In Hyogo Prefecture, a regional oncofertility network was established in 2016 as an organizational framework to facilitate collaboration between cancer treatment facilities and reproductive medicine centers. Regular meetings were initially held; however, these activities were gradually disrupted by the COVID-19 pandemic and personnel turnover. As a result, the network’s current activities have become largely limited to annual reporting, while educational programs and awareness initiatives have diminished. This experience suggests that establishing a network structure alone is insufficient to ensure sustainable operation. One of the major challenges was maintaining long-term interinstitutional collaboration. Although representatives were initially appointed at participating institutions, personnel turnover led to unclear points of contact and gradually weakened communication among participating facilities. Consequently, operational activities declined over time. These observations suggest that appointing institutional representatives is necessary but not sufficient for sustaining collaborative networks. Mechanisms that ensure continuity despite personnel changes are also essential. Because our institution does not have an in-house reproductive medicine department, it was not originally a central participant in the regional network. Nevertheless, given the substantial number of patients requiring oncofertility care and the recognized need for regular multidisciplinary communication, we established a dedicated collaborative framework with a geographically nearby reproductive medicine center. We believe that this partnership functioned effectively. Regular meetings were particularly valuable in facilitating smooth collaboration, and notably, web-based meetings proved to be sufficient for maintaining communication and coordination.

To address not only institutional challenges but also broader regional issues in oncofertility care, discussions aimed at revitalizing the regional network have recently been initiated among key stakeholders, including university hospitals and regional cancer centers. Current efforts focus on re-establishing clear points of contact at all cancer treatment hospitals within the prefecture. Because cancer care hospitals in Japan are required to maintain adolescent and young adult care teams, these teams may serve as appropriate and sustainable contact points for regional oncofertility networks. Relying on organizational units rather than individual physicians may improve continuity and reduce the impact of personnel turnover. Once contact points have been identified at each institution, regular meetings, educational seminars, and network-related information sharing are expected to resume. These experiences suggest that sustainable implementation requires not only an organizational framework but also continuous stakeholder engagement, clear communication channels, and regular opportunities for multidisciplinary interaction.

This implementation model could be readily adopted by neighboring hospitals, particularly when clear institutional contact points are established. Once responsible personnel or teams are identified within each participating institution, implementation of these tools is expected to be relatively straightforward. Furthermore, regular meetings between cancer treatment facilities and reproductive medicine centers would facilitate communication and allow practical issues to be addressed through ongoing collaboration and feedback. A potential barrier to broader implementation is the availability of personnel and institutional resources. Smaller hospitals may face challenges that differ from those encountered by university hospitals or large cancer centers with greater staffing capacity. Addressing these context-specific barriers will be important for sustainable implementation. Future regional meetings and network activities may provide opportunities to identify such challenges and develop practical solutions tailored to the needs of different institutions.

Knowledge and awareness of FP options also remain limited among both patients and healthcare providers. Reliance only on physician-led explanations during busy clinical practice increases the risk of missed opportunities and informational bias. Development of concise, structured information tools that support brief but effective counseling therefore represents an important priority. Prior studies identify time constraints, psychological burden, and unclear professional roles as major barriers to adequate FP counseling and referral (27, 28). Several factors may contribute to discontinuation after counseling, including concern about delaying cancer treatment, financial burden, psychosocial stress, and provider-side operational barriers (10, 12). In contrast, FP interventions do not necessarily delay cancer treatment initiation in some settings (21). These findings support the importance of transparent information sharing and rapid referral pathways to enable informed decision-making. In our cohort, almost all referred male patients underwent fertility preservation, whereas the proportion among female patients was approximately 70%. Previous studies have reported that up to 89% of patients who received a formal consultation with a reproductive specialist proceeded with some form of fertility preservation treatment (29). Another survey found that 73% of patients who received such consultations made a fertility preservation–related decision, which is consistent with our findings (30). In contrast, the rate of fertility preservation is reported to be lower when patients receive only general information or counseling without a formal reproductive consultation (31, 32). These findings suggest that timely referral to reproductive specialists may play an important role in supporting informed decision-making regarding fertility preservation. To facilitate patient understanding and support informed decision-making prior to formal consultation with reproductive specialists, we developed an informational leaflet for patients that provides information on fertility preservation options and financial assistance programs related to oncofertility care. The leaflet also includes QR codes that allow patients to easily access relevant websites and obtain additional information.

The strengths of this study include 4 years of accumulated data and demonstration of a stable, functioning support system across multiple cancer types and departments. Previous reports show that multidisciplinary oncofertility models improve provider awareness and referral practices (19, 20). Institution-wide frameworks with clearly defined referral pathways also strengthen interdepartmental collaboration, improve information sharing, and reduce practical and psychological barriers for clinicians (10, 33–35). At our institution, the WG functioned as a catalyst for institutional awareness and enabled rapid cross-specialty consultation.

Several challenges remain, including long-term follow-up of pregnancy and live birth outcomes after cryopreservation and support for patients who decline FP for psychological, financial, medical, or physical reasons (36). Future work should establish structured long-term follow-up systems and evaluate how FP support influences patient life planning and survivorship outcomes.

This study has several limitations. The single-center retrospective design and relatively small sample size limit generalizability. Furthermore, although the implementation model proved feasible at a regional cancer treatment cooperation base hospital without an in-house reproductive medicine unit, broader applicability likely depends on the availability, capacity, and geographic proximity of external partner reproductive centers. Moreover, long-term reproductive outcomes, including pregnancy and live birth after FP, were not adequately captured, which restricts evaluation of ultimate patient-centered endpoints. In addition, because patient-reported outcomes were not assessed, the impact of the program from the patient perspective could not be adequately evaluated. Future studies should investigate patient satisfaction, understanding of fertility preservation options, confidence in decision-making, and perceptions of the referral process.

## CONCLUSION

This study shows that even a regional cancer treatment cooperation base hospital without an in-house reproductive medicine unit can deliver timely and practical FP support through a multidisciplinary WG and structured collaboration with an external reproductive center. This approach represents a scalable and adaptable implementation model. Standardized referral pathways and CCS use transformed FP support from a physician-dependent activity into an institution-wide standardized system. Initial and follow-up questionnaires helped identify institution-specific barriers, measure the impact of the implemented framework, and define targets for further improvement in oncofertility care.

The short median interval of 2 days from referral to reproductive medicine consultation indicates that FP options can be offered without delaying cancer treatment initiation, which supports the clinical feasibility of this model. The framework relies more on structured coordination than on institutional size or geographic conditions and may therefore translate to other cancer centers without reproductive medicine units.

Future work should establish long-term follow-up systems for reproductive outcomes, including pregnancy and live birth, and maintain continuous education for both patients and healthcare providers. Sustainable program design aligned with regional resources and network-based oncofertility models will remain important for long-term success. These steps will strengthen FP service quality and help reduce regional disparities in oncofertility care through scalable implementation.

## Figures and Tables

**Figure 1:**
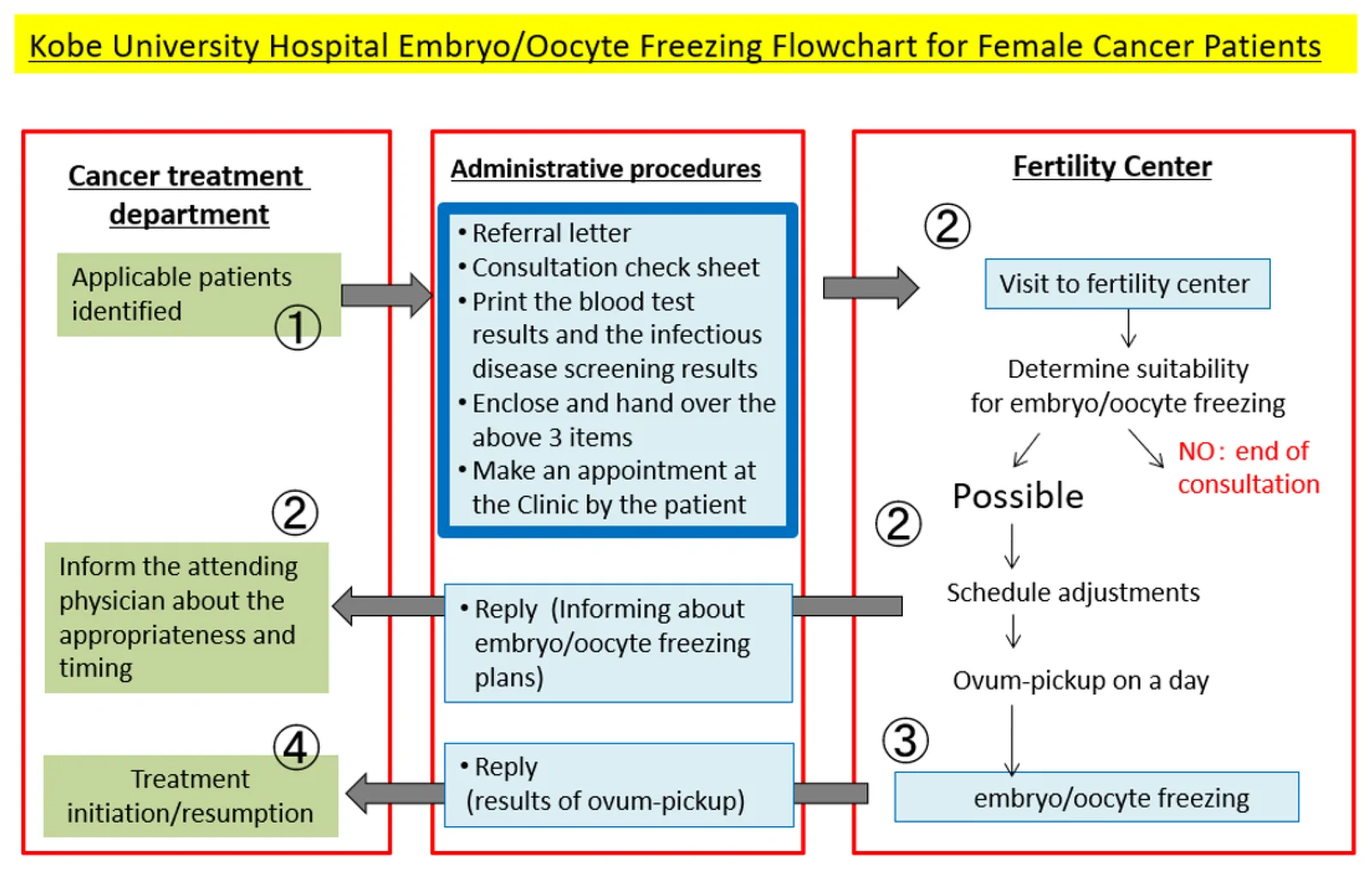
Flow chart

**Figure 2:**
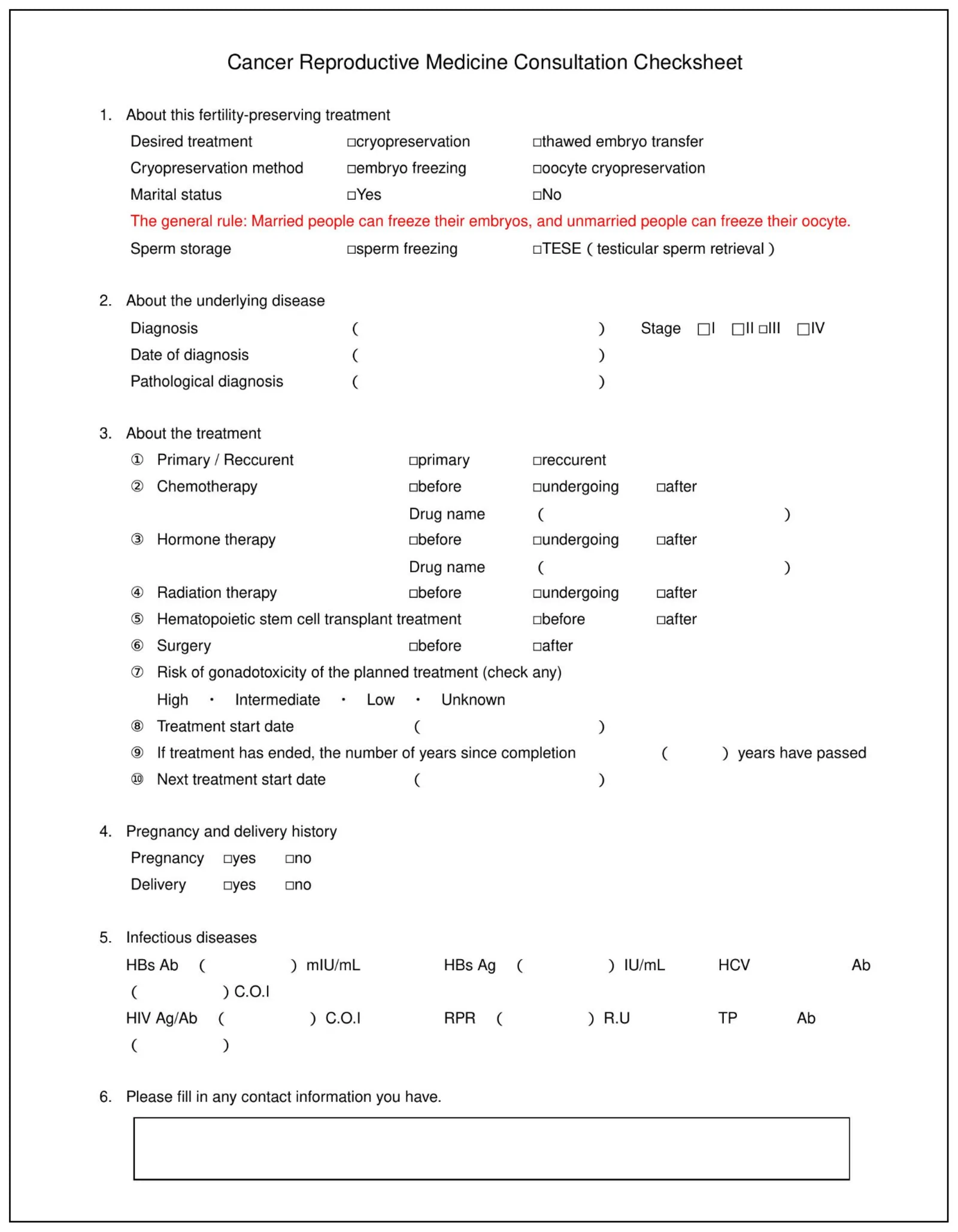
Consultation check sheet

**Figure 3:**
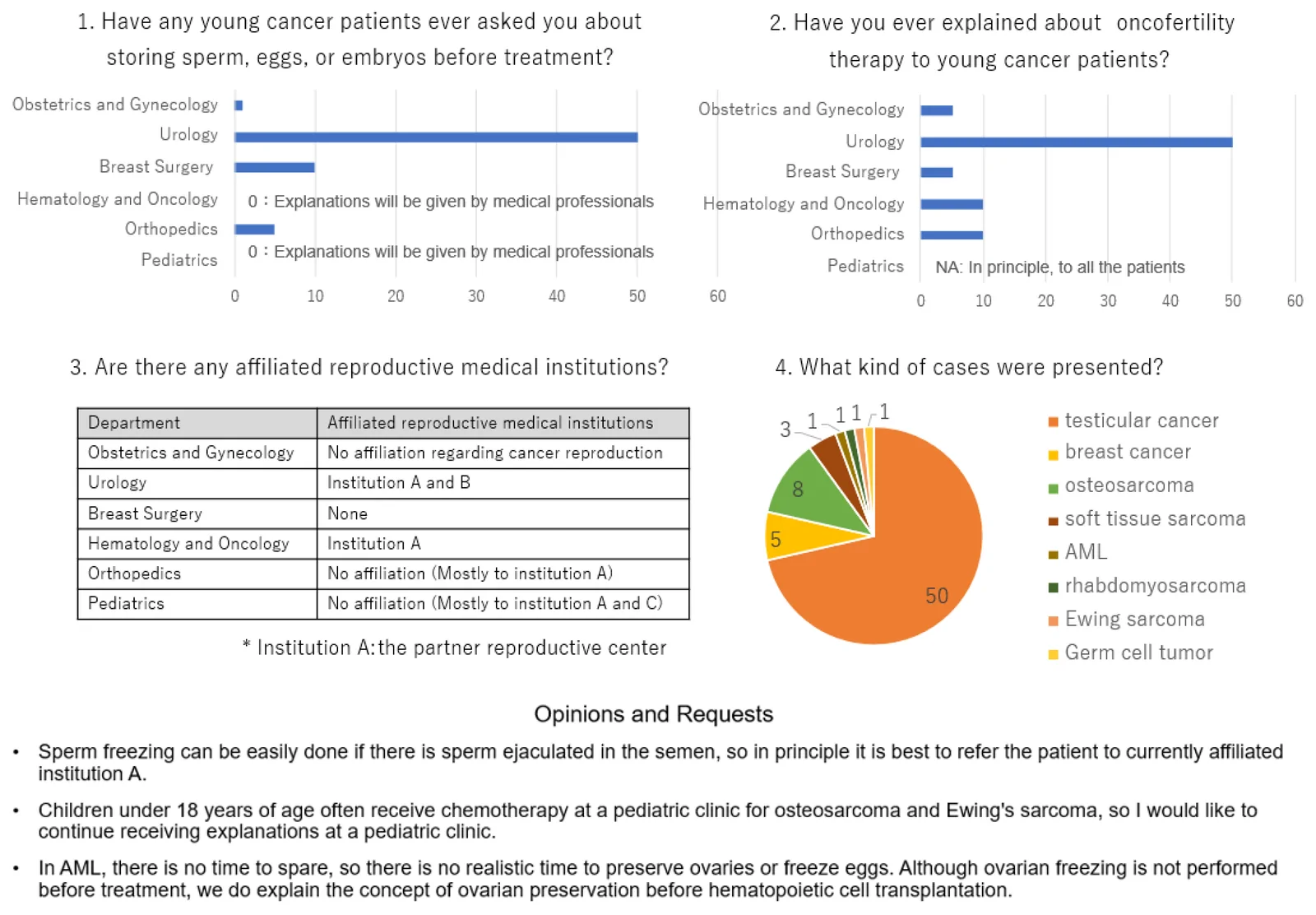
Result of initial questionnaire survey

**Figure 4:**
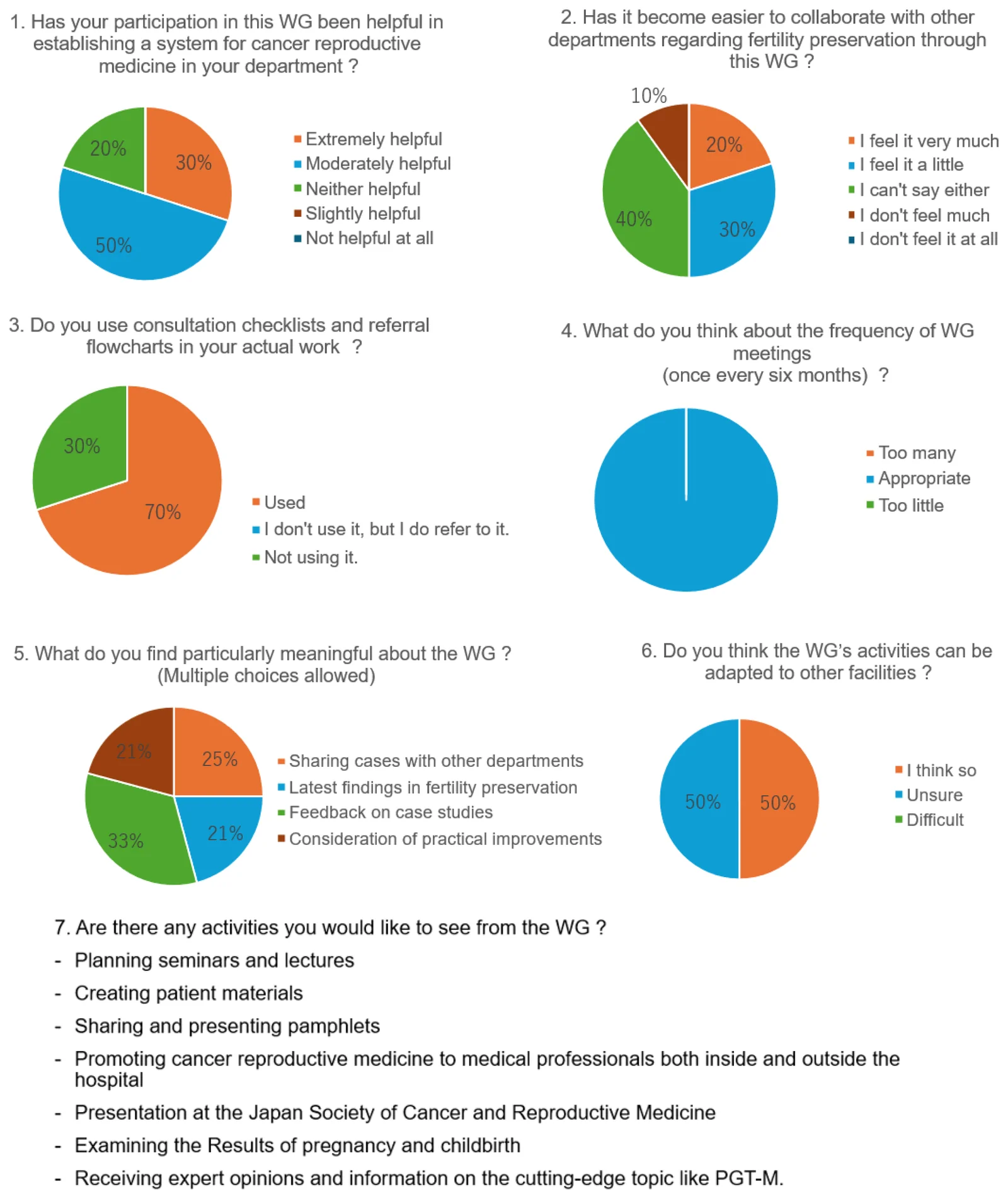
Result of follow-up questionnaire survey

**Table I:** Baseline characteristics

		Female (n = 24)	Male (n = 21)	Total (n = 45)
Age (years), median (range)		29 (16–42)	24 (9–47)	
Marital status	Unmarried	19 (42.2%)	17 (37.8%)	36 (80.0%)
	Married	5 (11.1%)	4 (8.9%)	9 (20.0%)
Number of children	Three	1 (2.2%)	0	1 (2.2%)
	Two	0	0	0
	One	2 (4.4%)	2 (4.4%)	4 (8.9%)
	None	21 (46.7%)	19 (42.2%)	40 (88.9%)

**Table II:** Referral characteristics

		Female (n = 24)	Male (n = 21)	Total (n = 45)
Referral year	2022	7 (15.6%)	4 (8.9%)	11 (24.4%)
	2023	8 (17.8%)	6 (13.3%)	14 (31.1%)
	2024	6 (13.3%)	6 (13.3%)	12 (26.7%)
	2025	3 (6.7%)	5 (11.1%)	8 (17.8%)
CCS status	With CCS	20 (44.4%)	17 (37.8%)	37 (82.2%)
	Without CCS	2 (4.4%)	4 (8.9%)	6 (13.3%)
	CCS completed after counseling	2 (4.4%)	0	2 (4.4%)
Referring department	Hematology and Oncology	13 (28.9%)	6 (13.3%)	19 (42.2%)
	Urology	0	8 (17.8%)	8 (17.8%)
	Pediatrics	0	5 (11.1%)	5 (11.1%)
	Breast and Endocrine Surgery	5 (11.1%)	0	5 (11.1%)
	Orthopedic Surgery	2 (4.4%)	0	2 (4.4%)
	Rheumatology/Collagen Disease Medicine	1 (2.2%)	0	1 (2.2%)
	Dermatology	1 (2.2%)	0	1 (2.2%)
	Obstetrics and Gynecology	1 (2.2%)	0	1 (2.2%)
	Respiratory Medicine	1 (2.2%)	0	1 (2.2%)
	Hepato-Biliary-Pancreatic Surgery	0	1 (2.2%)	1 (2.2%)
	Otolaryngology–Head and Neck Surgery	0	1 (2.2%)	1 (2.2%)
Days from referral to consultation	0	3 (6.7%)	1 (2.2%)	4 (8.9%)
	1	6 (13.3%)	4 (8.9%)	10 (22.2%)
	2	4 (8.9%)	3 (6.7%)	7 (15.6%)
	3	2 (4.4%)	3 (6.7%)	5 (11.1%)
	4	1 (2.2%)	0	1 (2.2%)
	5	0	1 (2.2%)	1 (2.2%)
	6	1 (2.2%)	3 (6.7%)	4 (8.9%)
	7–9	0	1 (2.2%)	1 (2.2%)
	10–19	2 (4.4%)	0	2 (4.4%)
	20–29	1 (2.2%)	0	1 (2.2%)
	30–59	0	0	0
	≥60	0	1 (2.2%)	1 (2.2%)
	Not available	2 (4.4%)	6 (13.3%)	8 (17.8%)

CCS, consultation checklist sheet.

**Table III:** Cancer type

	Female (n = 24)	Male (n = 21)	Total (n = 45)
Hematologic malignancy	9 (20.0%)	4 (8.9%)	13 (28.9%)
Bone and soft tissue cancer	2 (4.4%)	4 (8.9%)	6 (13.3%)
Breast cancer	5 (11.1%)	0	5 (11.1%)
Testicular cancer	0	4 (8.9%)	4 (8.9%)
Head and neck cancer	1 (2.2%)	3 (6.7%)	4 (8.9%)
Autoimmune disease	2 (4.4%)	1 (2.2%)	3 (6.7%)
Brain tumor	0	2 (4.4%)	2 (4.4%)
Colorectal cancer	1 (2.2%)	0	1 (2.2%)
Lung cancer	1 (2.2%)	0	1 (2.2%)
Skin cancer	1 (2.2%)	0	1 (2.2%)
Ovarian cancer	1 (2.2%)	0	1 (2.2%)
Aplastic anemia	0	1 (2.2%)	1 (2.2%)
Prostate cancer	0	1 (2.2%)	1 (2.2%)
Pancreatic cancer	0	1 (2.2%)	1 (2.2%)

**Table IV:** Gonadotoxicity risk

	Female (n = 24)	Male (n = 21)	Total (n = 45)
High	4 (8.9%)	8 (17.8%)	12 (26.7%)
Intermediate	10 (22.2%)	7 (15.6%)	17 (37.8%)
Low	7 (15.6%)	2 (4.4%)	9 (20.0%)
Unknown	3 (6.7%)	3 (6.7%)	6 (13.3%)
Not applicable	0	1 (2.2%)	1 (2.2%)

**Table V:** Overview of fertility preservation procedures

		Female (n = 24)	Male (n = 21)
Fertility preservation performed	Yes	17 (70.8%)	20 (95.2%)
	No (Consultation only)	7 (29.2%)	1 (4.8%)
Cryopreservation type	Embryo	2 (11.8%)	
	Oocyte	15 (88.2%)	
	Sperm		20 (100%)
Ovarian stimulation method	Antagonist protocol	10 (58.8%)	
	Antagonist protocol with aromatase inhibitor	1 (5.9%)	
	Progestin-primed ovarian stimulation	5 (29.4%)	
	Clomiphene citrate and in vitro maturation	1 (5.9%)	
Number of cycles	One cycle	13 (76.5%)	17 (85.0%)
	Two cycles	4 (23.5%)	3 (15.0%)
Oocyte outcomes, mean (range)	Oocytes frozen per cycle	8.2 (0–28)	
	Total oocytes frozen per patient	10.8 (1–28)	
Sperm outcomes, mean (range)	Vials frozen per cycle		5.3 (0–14)
	Total vials frozen per patient		6.1 (0–14)

**Table VI:** Clinical outcome

	Female (n = 24)	Male (n = 21)	Total (n = 45)
Alive at last follow-up	20 (83.3%)	17 (81.0%)	37 (82.2%)
Disease recurrence	3 (12.5%)	1 (4.8%)	4 (8.9%)
Death owing to primary disease	0	1 (4.8%)	1 (2.2%)
Transfer to another hospital	1 (4.2%)	2 (9.5%)	3 (6.7%)
